# Plateau hypoxia attenuates the metabolic activity of intestinal flora to enhance the bioavailability of nifedipine

**DOI:** 10.1080/10717544.2018.1469687

**Published:** 2018-05-23

**Authors:** Juanhong Zhang, Yuyan Chen, Yuemei Sun, Rong Wang, Junmin Zhang, Zhengping Jia

**Affiliations:** a School of Pharmacy, Lanzhou University, Lanzhou, China;; b Key Laboratory for Prevention and Remediation of Plateau Environmental Damage, Lanzhou General Hospital, Lanzhou, China

**Keywords:** Nifedipine, gut microbiota, metabolic activity, plateau hypoxia, bioavailability, pharmacokinetics

## Abstract

Nifedipine is completely absorbed by the gastrointestinal tract and its pharmacokinetics and metabolism may be influenced by microorganisms. If gut microbes are involved in the metabolism of nifedipine, plateau hypoxia may regulate the bioavailability and the therapeutic effect of nifedipine by altering the metabolic activity of the gut microbiota. We herein demonstrated for the first time that gut flora is involved in the metabolism of nifedipine by *in vitro* experiments. In addition, based on the results of 16S rRNA analysis of feces in rats after acute plateau, we first confirmed that the plateau environment could cause changes in the number and composition of intestinal microbes. More importantly, these changes in flora could lead to a slower metabolic activity of nifedipine in the body after an acute plateau, resulting in increased bioavailability and therapeutic efficacy of nifedipine. Our research will provide basis and new ideas for changes in the fecal flora of human acutely entering the plateau, and contribute to rational drug use of nifedipine.

## Introduction

The importance of the gut microbiome is speedy emerging in determining not only human health but also in the metabolism of drugs and xenobiotics (Yu et al., 2017). The immense number of microbes living in and around us is an important but largely underestimated component of therapeutics, stimulating efforts to supply pharmacologic and pharmacokinetic studies with detailed analyses of microbial communities (Haiser & Turnbaugh, 2012). The gut microbiota has both direct and indirect effects on drug and xenobiotic metabolisms, and this can have an effect on efficacy and toxicity (Wilson & Nicholson, 2017). Recent studies make it unambiguous that the human gut microbiota can play a vital role in the metabolism of xenobiotics, the stability and oral bioavailability of drugs. A mechanistic understanding of how the gut microbiota affects drug process *in vivo* is beginning to evolution. Any alteration to the gut microbiota could influence metabolic level of xenobiotics. As a typical example, high altitude environment is characterized by low pressure, hypoxia, strong radiation, coldness, etc. Hypoxia is the staple factor affecting the behavior and activities of human beings (Basnyat & Murdoch, 2003; Zafren et al., 2017). Hypoxia may alter the composition of the gut microflora (Sket et al., 2018), thereby affecting the biotransformation of drugs in the gastrointestinal tract before being absorbed (Jia et al., 2018).

Nifedipine, a dihydropyridin calcium channel, is mostly utilized for the clinical treatment of precordial angina, hypertension, and other vascular diseases (Yasam et al., 2016; Rubini et al., 2017). Importantly, nifedipine is a nonpolar drug that is completely absorbed by the gastrointestinal tract (Grigoriev et al., 2016; Filgueira et al., 2017). Its metabolism may be affected by microbiota in the stomach and intestine although the biotransformation of nifedipine by enteric microorganism has not yet been characterized. If gut microbiota are involved in the metabolism of nifedipine, the plateau hypoxia could modulate the bioavailability and therapeutic efficacy of nifedipine by altering the metabolic activity of the gut microbiota.

We herein attempt to prove whether the intestinal flora is involved in the metabolism of nifedipine in rats, and elucidate the effects of plateau hypoxia on the metabolic characteristics and pharmacokinetics of nifedipine by investigating the gut microbial metabolism, and eventually to demonstrate that plateau hypoxia could attenuate the metabolic activity of intestinal flora to enhance the bioavailability of nifedipine.

## Materials and methods

### Materials

The nifedipine tablets (lot no. F160504) were purchased from Shanxi Fenhe Pharmaceutical Co. Ltd. (China). The standard substance of nifedipine (lot no. 100338-201103) was obtained from China drug and biologic product standardization station (Beijing, PR China). The good merchantable quality dehydronifedipine (CAS number: 67035-22-7) was acquisitioned from Toronto Research Chemicals. Nimodipine (lot no. Y17A6C246g) was obtained from Shanghai YuanYe Biological Technology Co. Ltd. The formic acid, methanol, and acetonitrile were used chromatographically pure and purchased from Merck KGaA (Darmstadt, Germany). All other reagents use standardized reagents.

### Animal experiment

Male Wistar rats (weighing 180–220 g, certificate: SCXK (GAN) 2015-0001) were supplied by the Lanzhou Veterinary Research Institute. All animals were housed in rat box (six rats per cage) at 20–25 °C in a humidified atmosphere (50 ± 10%), and were fed standard laboratory diet and allowed water free. This study has been authorized by the Institutional Review Board of Lanzhou General Hospital of Lanzhou Military Command. All experiments were executed according to correlative guidelines and regulations.

They were divided into three groups: plain group of 1500 m in the city of Lanzhou (P), acute plateau group of 4100 m in the Yushu autonomous prefecture of Qinghai province (H), and amoxicillin-administered plain group of 1500 m in the city of Lanzhou (P1) (*n* = 12 for each group). The H group was transported by bus from Lanzhou to Yushu autonomous prefecture of Qinghai province of 14 h and involved trip about 900 km. For P1 group, rats were administered amoxicillin (157.5 mg/kg) orally once a day for 2 days.

### 16S rRNA analysis

After 9 h of fasting, rat stool samples were collected in plastic cups and frozen immediately at –80 °C until analysis. 16S rRNA analysis was performed to research the alterations in gut microbiota. After mixing the feces of 12 rats in each group, three aliquots were taken for 16S rRNA analysis. Frozen fecal samples from rats were diluted 1:10 (w/v) in phosphate buffer and thawed at 4 °C. DNA was extracted and then subjected to PCR (Leser et al., 2000). The bioinformatics analysis will be carried out with sequencing data. The original data were filtered to remove low-quality information and the tags were clustered into operational taxonomic units (OTUs) with 97% sequence similarity. Taxonomic ranks were assigned to OTU emblematic list using Ribosomal Database Project (RDP) Naive Bayes Classifier v.2.2. At last, alpha diversity, beta diversity, and the different species screening were analyzed.

### Stool extracts preparation

Rat fecal specimens (about 0.5 *g* each) were suspended in 4.5 ml of cold saline, and then the suspension liquid was centrifuged at 500 *g* for 5 min. The resulting supernatant was sonicated for 10 min and then centrifuged at 10,000 *g* for 20 min. The resulting supernatant was employed to metabolic rate assessment (Kim et al., 2016).

### Assay of nifedipine-metabolizing activity and metabolites in the intestine

The reaction mixture system, including 0.1 ml of excrement extract, 0.1 ml of nifedipine (1.44 mM), and 0.3 ml of 0.1 M phosphate buffer (pH 7.0), was incubated at 37 °C for 12 and 24 h (Kim, 2015; Yoo et al., 2016). Next, 950 μl of frozen acetonitrile was added to the incubation system (50 μl) to stop the reaction, and 30 μl of the mixture was pipetted into the centrifuge tube. Each tube was vortexed for 1 min for extraction, followed by centrifugation for 5 min at 13,000 *g*. The organic stratum was then transferred to a clean sample bottle, and 10 μl specimens was introduced into the ultra-fast liquid chromatography/tandem mass spectrometry (UFLC-MS/MS) system for analysis of nifedipine and oxidation nifedipine. The remaining supernatant specimen was used for metabolite identification by MS/MS.

### Pharmacokinetic experiments

Pharmacokinetic experiments were performed 2 days after the last dose. The treatment was the same for all three groups: overnight after fasting, each rat was given a single oral nifedipine (1.05 mg/kg) dissolved in 2 ml of water. Blood samples were collected from veins of eye socket into heparinized centrifuge tubes at 0.167, 0.33, 0.5, 0.75, 1, 1.5, 2, 4, 6, 8, 12, and 24 h after drug administration. The whole blood samples were centrifuged at 3000 × *g* for 5 min to get adtevak and then stored at −20 °C. All adtevak samples from the three groups were transported to the Lanzhou laboratory where they were then stored at −20 °C until the quantitative analysis.

### Sample preparation

Compounds were extracted from the biologic sample using albumen precipitation method. The sample was thawed to room temperature, and an aliquot of 25 ng/ml nimodipine (internal standard in 75 µl acetonitrile) was added to 30 µl of the collected sample in a 0.5 ml centrifuge tube. The tubes were vortexed mixed for 1 min. After centrifuging at 13,000 *g* for 5 min, the supernatant was injected onto the HPLC column for UFLC-MS/MS analysis.

### UFLC-MS/MS analysis

For analysis of the nifedipine and metabolites of nifedipine, UFLC-MS/MS analyses were performed on the UFLC composed of a fully outfit Agilent 1100 (CA, USA). Detection was executed on an API 3200 MS system triple quadrupole mass spectrometer from Applied Biosystems controlled by Analyst 1.4 software (CA, USA) equipped with Shim-pack XR-ODS column (3.0 mm I.D.×75 mm).

Plasma concentration and excretion of nifedipine and nifedipine were determined by electrospray ionization. The mobile phase was acetonitrile–water (90:10, v/v). For beamed MS/MS analysis, the product ion scan area was *m/z* 50–400. High-purity nitrogen was recommended as fragmentation gas. The ion source parameters were investigated as the following: Ion spray (IS) voltage was –4500 V, ion source temperature (TEM) was 300 °C. Quantitation was carried out using the multiple reactions monitoring (MRM) to study precursor → product ion transitions were *m/z* 345.0 → 122.0 for nifedipine and 417.0 → 122.0 for nimodipine. Collision energy (CE) were –19 eV and –30 eV, declustering potential (DP) were –21 V and –57 V, respectively. Quantitation was performed for oxidation nifedipine was *m/z* 345.0 → 122.0, IS was 4500 V, TEM was 300 °C, CE was 38 eV, and DP was 50 V.

### Data analysis

Software DAS 2.0 was used to analyze pharmacokinetic data of nifedipine. All data were showed as mean ± standard deviation (SD). Analysis of statistical significance was performed, and *p* < .05 was considered to manifest a statistically significant result. Statistical analysis was done using SPSS version 13.0 software.

## Results

### Effects of Plateau hypoxia on intestinal microflora

#### Stool microscopic observations

**Figure 1.**
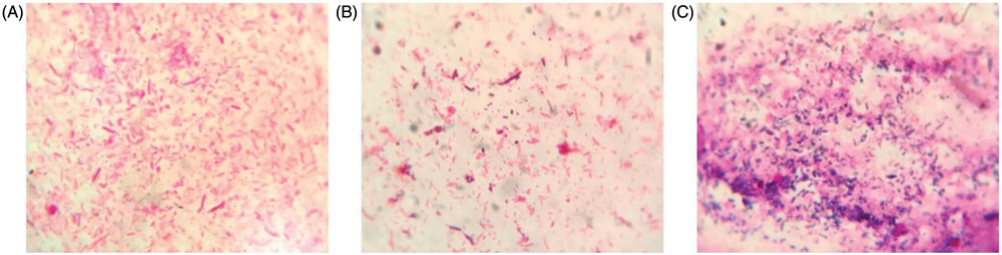
Examining the stool smear under the microscope of A: plain group, B: high altitude group, and C: amoxicillin-administered plain group.

To characterize the effects of plateau hypoxia on gut microbiota, stool samples were smeared and stained and the consequent changes in the number of representative gut microbiota were observed microscopically. As shown in Figure 1, amoxicillin treatment group as a control group, plateau group appeared *Bacillus*. Compared with the normal (plain) rats, amoxicillin treatment group significantly reduced the number of *Enterobacteriaceae*. Our results indicate that plateau hypoxia and amoxicillin treatment could alter the gut flora.

#### 16S rRNA

**Figure 2.**
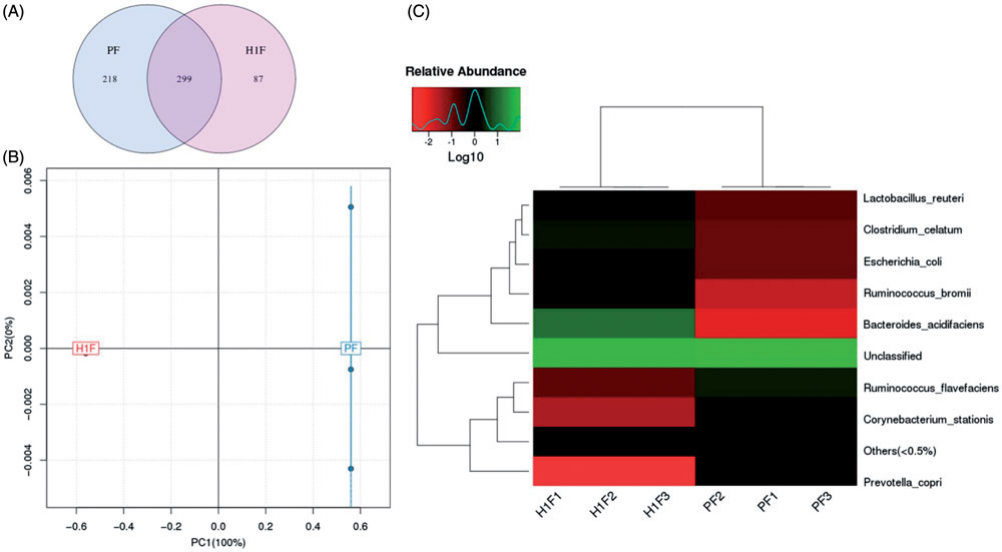
16S rRNA analysis results (PF: fecal samples of plain group, H1F: fecal samples of hypoxia group.) of A: Venn diagram was shared OTU across different groups, B: PCA based on OTU abundance between two groups, C: Log-scaled percentage heat map of species level.

The 16S rRNA fragment was used to analyze intestinal microflora of P and H groups. Alpha diversity is used to analyze the complexity of species diversity by a number of indicators including the observed species Chao1, ACE, Shannon, and Simpson (Schloss et al., 2009). Table 1 shows the statistics of alpha diversity. The Sobs, Chao, ACE, and Shannon values of the plateau rats were significantly lower than those of the plain group, and Simpson was significantly increased. These results indicate that the plateau environment significantly reduces the number and variety of intestinal flora. Venn diagram can vividly show the number of corporate or particular OTUs in different groups. If combined with OTU representatives of species, the kernel microbiomes of different environments could be obtained. Light blue (218) is a plain group and Light pink (87) is a plateau group. The overlapping area of lavender (299) represents the set of OTU commonly present in the counterpart samples (Figure 2(A)). These results show that the composition of the flora was different between the two groups, and plain group rats had more abundant flora. To display the differences of OTU composition in different samples, principal component analysis (PCA) was used to summarize factors mainly dependable for these differences, and the similarity is high when two samples are close to the location. Based on the OTU abundance information, the relative abundance of each OTU in each sample would be calculated, and the PCA of OTU was done with the relative abundance value, blue represents plain group and red represents plateau group (Figure 2(B)). There was an obvious separation between two groups. Plain rats were characterized by positive scores, whereas plateau rats exhibited negative scores on PC1. Heat maps are graphical representations of data in which the individual values contained in the matrix are represented as colors. Here, the heat map shows species clustering based on the abundance of each species. Horizontal clustering indicates the similarity of some species among different samples. Vertical clustering shows the similarity of all species across different samples. Species heat map analysis is based on the relative abundance of each species in each sample. As shown in Figure 2(C), there were differences in gut microbiome composition between the two groups. At the species level, the number of *Bacteroidetes* increased significantly and the number of *Prevotella* decreased at high altitude group. These results show that the plateau environment could lead to changes in the fecal flora.

**Table 1.** Alpha diversity statistics.

Sample name	Sobs	Chao	Ace	Shannon	Simpson	Coverage
PF1	517	590.615	578.468	4.782	0.017	0.996
PF2	517	591.820	576.922	4.782	0.018	0.996
PF3	517	593.560	577.991	4.780	0.018	0.996
H1F1	386	452.978	453.179	3.928	0.059	0.996
H1F2	386	451.553	453.264	3.928	0.059	0.996
H1F3	386	451.553	453.264	3.929	0.059	0.996

PF: fecal samples of plain group, H1F: fecal samples of hypoxia group.

### Determination of fecal and plasma concentration

**Figure 3.**
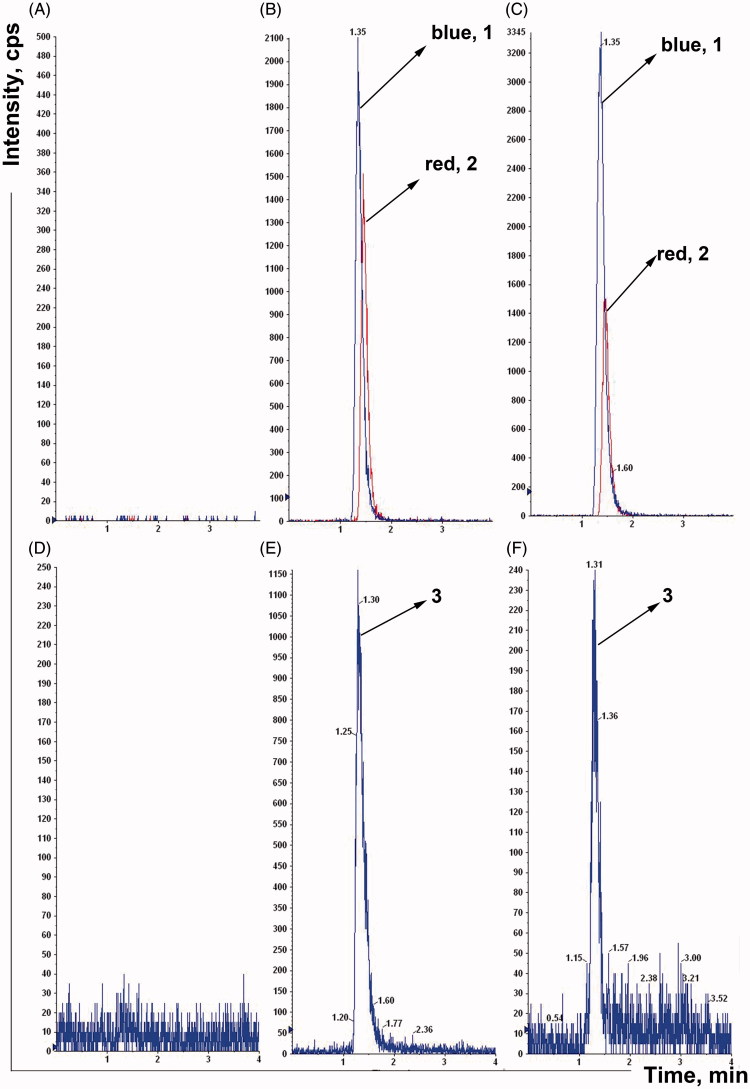
The chromatograms of nifedipine and oxidation nifedipine in rat stool samples (A, B, and C were nifedipine, D, E, and F were oxidation nifedipine. 1, nifedipine, 2, nimodipine, 3, oxidation nifedipine). A: Blank fecal fluid, B: blank fecal fluid spiked with standard nifedipine, C: rat stool samples were incubated for 12 h, D: blank fecal fluid, E: blank fecal fluid spiked with standard oxidation nifedipine, and F: rat stool samples were incubated for 12 h.

To determine whether the intestinal microflora is involved in the metabolism of nifedipine and the effects of plateau hypoxia on intestinal microflora-mediated nifedipine metabolism, the rats were transported rapidly to the plateau, and nifedipine-metabolizing activity was measured with the fecal samples collected from plateau hypoxia animals. Fecal samples were collected from the plain, plateau hypoxia and amoxicillin-treated rats, and the nifedipine metabolic characteristics and metabolizing activity of each group was examined. The chromatograms of nifedipine and oxidation nifedipine were depicted in Figure 3(A–F). The retention times of nifedipine, nimodipine, and oxidation nifedipine using our system were 1.35, 1.45, and 1.30 min, respectively. No significant interfering peaks observed in the biologic matrixes. It showed that the method exhibited good specificity and selectivity and was applicable to stool samples analysis.

### Biotransformation by rat fecalases and metabolizing activity of nifedipine

**Figure 4.**
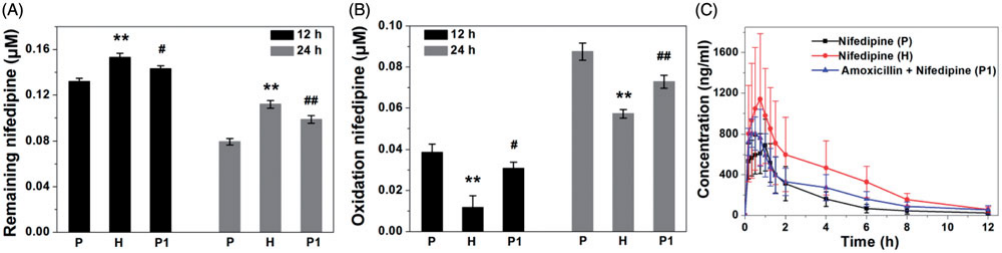
Nifedipine-metabolizing activities of rat fecal samples (P: plain rats; H: plateau hypoxia rats; P1: amoxicillin-treated rats.). A: the remaining amount of nifedipine after incubation for 12 and 24 h, B: formation of the oxidation nifedipine following incubation for 12 and 24 h. Data are expressed as mean ± standard deviation. C: Mean plasma concentration–time curve of nifedipine in rats at three groups. *p < .05, **p < .01 comparing with P. #*p* < .05, ##*p* < .01 comparing with P.

Nifedipine was incubated with rat fecal suspension for 12 and 24 h, and the remaining amount of nifedipine was measured by UFLC-MS/MS. As shown in Figure 4(A), the remaining amount of nifedipine was decreased after 12- and 24-h incubation in three groups, indicating the metabolic clearance of nifedipine by fecal microbial enzymes. Based on these data, the metabolic nifedipine percentage after incubation 12 h were 23.14% at plain group, 10.84% at high altitude group, and 16.67% at amoxicillin-treated group, and the levels of nifedipine were reduced by 53.72, 34.79, and 42.57% after incubation for 24 h, respectively, compared with their corresponding samples at 0 h. The experiment proved that the intestinal flora really related in the metabolism of nifedipine and biotransformation. In particular, rat fecal samples of three groups exhibited a large variation in nifedipine-metabolizing activity. The oxidation nifedipine was found to be a major metabolite generated by internal metabolism, the formation of oxidation nifedipine was measured in the fecal samples to confirm the correlation with the metabolic clearance of the parent drug. As shown in Figure 4(B), the oxidation nifedipine formation increased gradually, depending on the incubation time of the stool samples, which was consistent with the pattern of the loss of the parent drug. Plateau hypoxia and oral administration of amoxicillin prominently reduced nifedipine-metabolizing activity compared with plain rats. This result indicated that the metabolism of nifedipine was inhibited by suppression of the metabolic activity of gut microbiota following plateau hypoxia and amoxicillin treatment, and also confirmed the involvement of gut microbiota in the metabolism of nifedipine.

### Identification of nifedipine metabolites generated by fecal suspension and MS/MS

**Figure 5.**
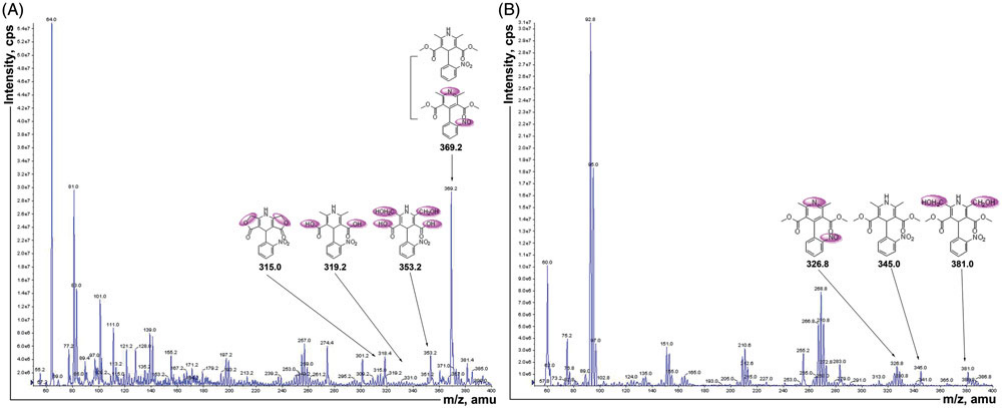
The mass spectrum of nifedipine by MS/MS. A: positive ion scan after incubation 12 h, B: negative ion scan after incubation 12 h.

Since changes in the gut microbiota caused by plateau hypoxia affect the metabolism of nifedipine, we next identified the metabolites of nifedipine. The rat fecal suspension sample was analyzed using MS/MS to identify the metabolites generated from nifedipine by intestinal flora. The fragmentation pattern of the MS/MS parent ion spectrum of M1 to M6 (Figure 5(A,B) was consistent with the literature except M6 (Raemsch & Sommer, 1983; Ramsch et al., 1986; Soons et al., 1991). Mass spectrometric data indicated that six metabolites were detected and M1 (major) is oxidation nifedipine at *m/z* 344.10 (C_17_H_16_N_2_O_6_). The study also found mesostate of M6 at *m/z* 350.08 (C_15_H_14_N_2_O_8_). M2 to M5 were *m/z* 328.11 (C_17_H_16_N_2_O_5_), *m/z* 318.09 (C_15_H_14_N_2_O_6_), *m/z* 378.11 (C_17_H_18_N_2_O_8_), and *m/z* 314.05 (C_15_H_10_N_2_O_6_). This finding also suggested that gut microbiota is involved in the conversion of nifedipine to its metabolite. Thus, any changes in intestinal flora could lead to the alteration in its metabolic activity to affect the nifedipine metabolite formation in the intestinal.

### Effects of plateau hypoxia on the pharmacokinetics of nifedipine

Subsequently, to investigate the effects of plateau hypoxia on nifedipine pharmacokinetics, the plasma levels of nifedipine were determined following oral administration of nifedipine at three group rats. Nifedipine was administered 3 days after the antibiotic treatment to rule out any pharmacokinetic interactions of antibiotics with drug transporters or cytochrome P450 enzymes. This study found significant changes in the pharmacokinetics of nifedipine under the special environment of high altitude hypoxia. The concentration–time profiles in plasma obtained from the three groups have similar shapes (Figure 4(C)). There were no statistically significant differences of pharmacokinetics parameters between the group H and group P1. The area under the concentration curve (AUC) and the mean residence time (MRT) obtained an increase in group H, whereas plasma clearance (CL) was lower in rats, compared with the group P. The AUC was significantly increased 39.10% in group P1, and the peak time (Tmax) and CL were decreased 48.91% and 34.71%, compared with the group P. There were no statistically significant differences in other parameters between the three groups.

## Discussion

In the present work, we aimed at characterizing the intestinal microbiota composition at acute high altitude rats, and to investigate the effect of plateau hypoxia-mediated intestinal flora dysregulation on the metabolic conditions and bioavailability of nifedipine. First, we observed the effects of plateau hypoxia on gut microbiota by examining the stool smear under the microscope. The metabolic activity of the rat fecal suspension was significantly reduced upon rapid entry into the plateau (Figure 1). Second, the 16S rRNA fragment was used to analyze intestinal microflora of rat stool samples (Figure 2). Eventually, we demonstrated that the plateau hypoxia could lead to changes in the fecal flora, which further confirms that the environment could change the intestinal flora (Jia et al., 2018; Sket et al., 2018).

Like the host transporters and metabolic enzymes, gut microorganisms actively participate in determining the efficacy, bioavailability, and side effects of orally administered drugs (Swanson, 2015). The intestinal flora plays a very important role in nifedipine metabolism although the liver is the major metabolic sites (Gandhi et al., 2013). We found that the intestinal flora plays an extensive role in nifedipine metabolism and in this manner contributes to the host *via* its impact on the absorption, metabolism, and maintenance of intestinal barrier function. First of all, we have established a specific and selective method to assay the nifedipine and oxidation nifedipine in rat stool samples (Figure 3). Next, we used the method established above to assess the metabolic activity of nifedipine and oxidation product changes after incubation of nifedipine with rat feces (Figures 4 and 5). Finally, we demonstrated that the effect of plateau hypoxia-mediated intestinal flora dysregulation on the bioavailability of nifedipine. Many studies have proved antibiotic administration to have both short- and long-term effects on intestinal flora composition in animals and humans. In particular, changes in intestinal microflora following antibiotic treatment have an effect on the metabolism of the drugs *in vivo* (Dethlefsen & Relman, 2011). Therefore, we employed amoxicillin pretreated rats as a control throughout the entire experiment, and we obtained the same results.

The gut microbiome that affects the absorption, metabolism, disposition toxicity, and pharmacology of drugs has been identified (Koppel et al., 2017). Special environment will cause flora imbalance, which in turn may affect the metabolism of drugs in the body. It is clear that microbial activities can alter the bioavailability and toxicity of drugs, as well as extend to evaluate the safety of drugs. The effects of the gut microbiota may include the modulation of host metabolic enzymes or transporters, direct competition for metabolism *via* particular host metabolic routes or enzymes as a result of other effects on host biochemistry (Wilson & Nicholson, 2017). We need to develop more advanced experimental approaches to define more completely factors and mechanisms on drugs metabolism. These advances will not only allow us to improve our ability to predict an individual’s response to specific drugs but also provide new opportunities for exploiting the host–microbiome relationship to develop either more effective or safer therapies. According to the clinical trial reports on nifedipine, the incidence of side effects of nifedipine occurred in a dose-related manner. Elevated plasma levels of nifedipine might increase the frequency of those side effects at high altitude. These pharmacokinetic issues could be expanded to include cases of bowel malfunction with abnormal microflora. Therefore, any conditions accompanied by alterations in gut microflora should require caution, and if necessary, nifedipine dose adjustment following clinical monitoring could be recommended at high altitude or antibiotics combined.

## Conclusion

In conclusion, we have first demonstrated that nifedipine could be metabolized by gut microbes, and the plateau environment could affect the metabolism activity, pharmacokinetics, and bioavailability of nifedipine due to intestinal microbial changes. The effect of plateau hypoxia-mediated intestinal flora dysregulation on the bioavailability of nifedipine in rats provides deep insight into the understanding of how this drug acts in human body, and this novel discovery may provide certain reference significance for the clinical use of nifedipine.
